# An App Developed for Detecting Nurse Burnouts Using the Convolutional Neural Networks in Microsoft Excel: Population-Based Questionnaire Study

**DOI:** 10.2196/16528

**Published:** 2020-05-07

**Authors:** Yi-Lien Lee, Willy Chou, Tsair-Wei Chien, Po-Hsin Chou, Yu-Tsen Yeh, Huan-Fang Lee

**Affiliations:** 1 Department of Medical Affairs Chi Mei Medical Center Tainan Taiwan; 2 Department of Information Management and Institute of Healthcare Information Management National Chung Cheng University Chayi Taiwan; 3 Department of Physical Medicine and Rehabilitation Chiali Chi Mei Hospital Chi Mei Medical Groups Tainan Taiwan; 4 Department of Physical Medicine and Rehabilitation Chung Shan Medical University Taichun Taiwan; 5 Department of Medical Research Chi Mei Medical Center Chi Mei Medical Groups Tainan Taiwan; 6 Department of Orthopedics and Traumatology Taipei Veterans General Hospital Taipei Taiwan; 7 School of Medicine National Yang-Ming University Taipei Taiwan; 8 Medical School St George’s, University of London London United Kingdom; 9 Department of Nursing College of Medicine National Cheng Kung University Tainan Taiwan

**Keywords:** nurse burnout, MBI-HSS Chinese version, receiver operating characteristic curve, convolutional neural network, Lz person fit statistic

## Abstract

**Background:**

Burnout (BO), a critical syndrome particularly for nurses in health care settings, substantially affects their physical and psychological status, the institute’s well-being, and indirectly, patient outcomes. However, objectively classifying BO levels has not been defined and noticed in the literature.

**Objective:**

The aim of this study is to build a model using the convolutional neural network (CNN) to develop an app for automatic detection and classification of nurse BO using the Maslach Burnout Inventory–Human Services Survey (MBI-HSS) to help assess nurse BO at an earlier stage.

**Methods:**

We recruited 1002 nurses working in a medical center in Taiwan to complete the Chinese version of the 20-item MBI-HSS in August 2016. The k-mean and CNN were used as unsupervised and supervised learnings for dividing nurses into two classes (n=531 and n=471 of suspicious BO+ and BO−, respectively) and building a BO predictive model to estimate 38 parameters. Data were separated into training and testing sets in a proportion 70%:30%, and the former was used to predict the latter. We calculated the sensitivity, specificity, and receiver operating characteristic curve (area under the curve) across studies for comparison. An app predicting respondent BO was developed involving the model’s 38 estimated parameters for a website assessment.

**Results:**

We observed that (1) the 20-item model yields a higher accuracy rate (0.95) with an area under the curve of 0.97 (95% CI 0.94-0.95) based on the 1002 cases, (2) the scheme named matching personal response to adapt for the correct classification in model drives the prior model’s predictive accuracy at 100%, (3) the 700-case training set with 0.96 accuracy predicts the 302-case testing set reaching an accuracy of 0.91, and (4) an available MBI-HSS app for nurses predicting BO was successfully developed and demonstrated in this study.

**Conclusions:**

The 20-item model with the 38 parameters estimated by using CNN for improving the accuracy of nurse BO has been particularly demonstrated in Excel (Microsoft Corp). An app developed for helping nurses to self-assess job BO at an early stage is required for application in the future.

## Introduction

### Burnout in the Workplace

Burnout (BO) is a critical syndrome and problem in high-tech service-oriented societies, particularly for nurses in health care settings [1-4]. Many studies [5-11] reported that BO influences an employee’s physical and psychological status [5-7], the organizational well-being [8-11], and patient quality-of-care outcomes [6,10].

One of the most popular BO inventories is the Maslach Burnout Inventory–Human Services Survey (MBI-HSS) [12,13]. More than 1898 articles were found by searching the keywords “Maslach” and “burnout” on September 23, 2019. BO is defined by Maslach [12,13] as a syndrome of emotional exhaustion, reduced personal accomplishment (PA), and depersonalization that frequently occurs in individuals who work in people-related jobs, such health care and educational.

### Maslach Burnout Inventory–Human Services Survey

The MBI-HSS [13] has been widely applied to measure individual BO in numerous workplaces [4,11,14-16]. The original MBI-HSS is a 22-item inventory with a 7-point scale (from never=0 to every day=6) to measure BO for workers in a recent week [13]. The three BO subscales comprise 9 items for emotional exhaustion, 8 items for personal accomplishment, and 5 items for depersonalization. Despite the survey being popularly used in social science, the cutting point for determining BO substantially differs between cultures and health care settings [15,17-21]. Accordingly, Maslach et al [22] suggested that BO levels (low, moderate, and high) had different cutting points in different countries and areas. Schaufeli and Van Dierendonck [23] suggested having common cutting points to compare BO levels among countries and areas.

Maslach and Jackson [13] suggested that the cutting points be set at 54 for emotional exhaustion, 48 for personal accomplishment, and 30 for depersonalization using subscale scores for measurement. Schaufeli and Van Dierendonck [23] were critical of the fact that the scheme for determining BO levels was arbitrary based on the three groups that contained an equal number of sample sizes [24]. Although Maslach and Jackson [13] also suggested having valid criteria that can be used for classifying BO levels, no such reasonable and viable scheme has been accepted by practitioners in the past.

### Convolutional Neural Network

Convolutional neural network (CNN) has had the greatest impact within the field of health informatics [25]. Its architecture can be described as an interleaved set of feedforward layers implementing convolutional filters followed by reduction, rectification, or pooling layers [26-28]. For each layer, the CNN creates a high-level abstract feature. Whether the CNN, a famous deep learning method, can improve the prediction accuracy (up to 7.14%) [28] on nurse BO classification is worthy of study.

### Online Classification Using Smartphones is Required

As with all forms of web-based technology, advances in mobile health communication technology are rapidly increasing [29]. Until now, there has been no app for smartphones to classify nurse BO levels. If the CNN BO model’s parameters have been estimated by the CNN algorithm, the classification of nurse BO by responding to the MBI-HSS can alert individual nurses more accurately and warn them to alleviate their mental strain before it becomes a serious BO problem.

### Study Aims

The aims of our study are to (1) estimate the model’s parameters using CNN based on nurse responses to the MBI-HSS and (2) design an app for smartphones based on a website assessment of nurse BO.

## Methods

### Data Source

#### Study Sample and Demographic Data

If the confidence level and intervals were set at 0.05 and ±5% and applied to the population of 1850 registered nurses in a hospital, 318 participants are required for the sample size [30]. We estimated the rate of refusal to respond will reach 40%. The minimum number for the study sample size will be 540 (318/[1–0.4]).

We delivered 40 copies each of the MBI-HSS BO survey to 32 nursing units. A sample of 1255 registered nurses with at least 1 month experience in the Chi Mei Medical Center (Taiwan) was randomly selected to complete the Chinese version of the 20-item MBI-HSS [3] in August 2016. A total of 1002 participants were eligible, for a return rate of 79.9%.

#### Featured Variables

Featured variables consist of the 20 items (called the 20-item model in which the response in the subscale of reduced personal accomplishment has been reversed to be the higher score denoting the more serious BO problem) on the classification of nurse BO levels (ie, suspicious BO+ and BO−). The 1002 participants were split into training and testing sets in a proportion (70%:30%), and the former was used to predict the latter. The data are shown in Multimedia Appendix 1. This study was approved and monitored by the Chi Mei Medical Center institutional review board (10704-003). All hospital and study participant identifiers were stripped.

### Unsupervised and Supervised Learnings

Unsupervised learning indicates agnostic aggregation of unlabeled data sets yielding groups or clusters of entities with shared similarities that may be unknown prior to the analysis step [31,32] (eg, clustering dimensionality reduction using principle component analysis or k-mean clustering). The k-mean clustering aims to partition n observations into k clusters, in which each observation belongs to the cluster with the nearest mean [33]. In contrast, supervised learning employs labeled training data sets (labeled/supervised by subject experts or by the objective k-mean clustering) to yield a qualitative or quantitative output [31,34].

In this study, the k-mean was used as unsupervised learning for clustering participants into two classes (n=531 and n=471 for suspicious BO+ and BO−, respectively). CNN was applied as supervised learning to build a BO prediction model for estimating the 38 parameters.

### Convolutional Neural Network Applied in This Study

**Figure 1:**
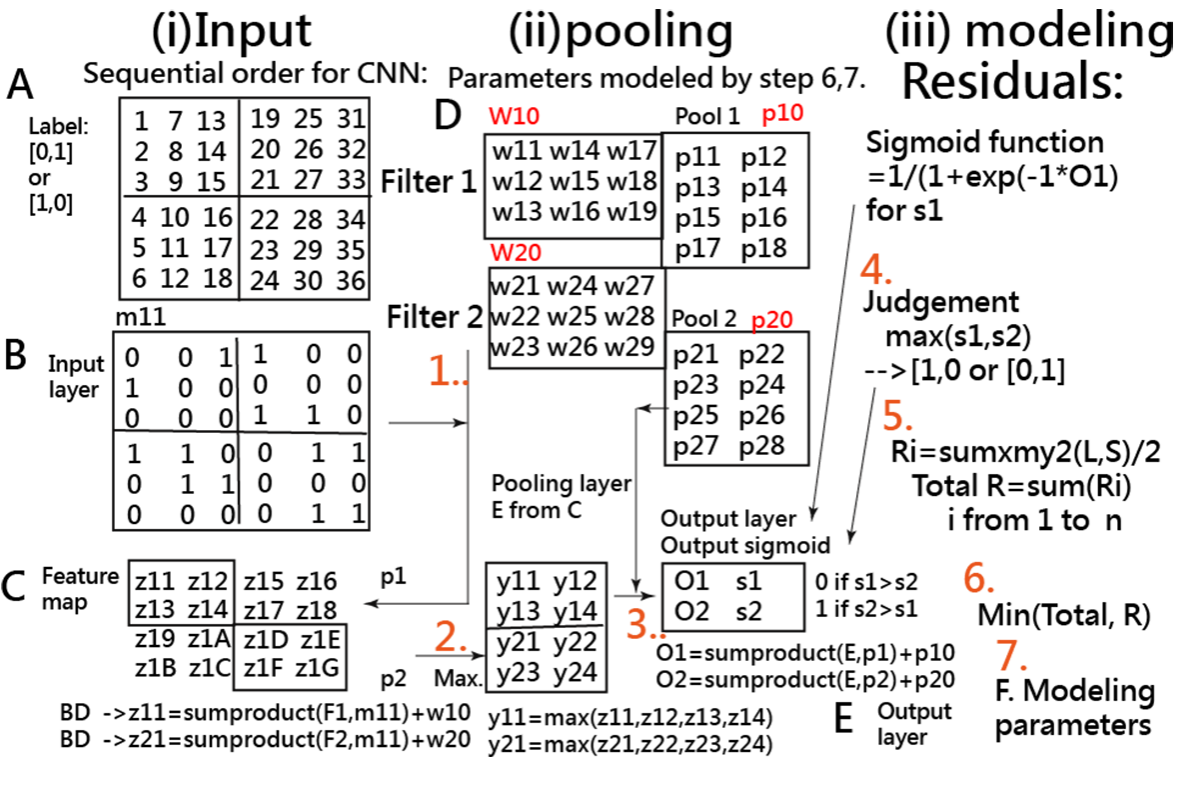
Interpretation of the convolutional neural network algorithm.

CNN is a variant of the standard multilayer perceptron, especially used for pattern recognition compared with conventional approaches [35] due to its capability in reducing the dimension of data, extracting the feature sequentially, and classifying one structure of the network [36]. The basic CNN model was inspired in 1962 from the visual cortex proposed by Hubel and Wiesel [35]. For simplifying the CNN concept and process, we present it in Figure 1. Detailed information on interpretation is provided in Multimedia Appendix 2.

### Tasks for Performing Convolutional Neural Network

#### Task 1: Comparison of Prediction Accuracies in Two Modes

Two sets of featured variables (ie, 20 with the traditional accurate rate and 100% rate) on 1002 cases were mirrored to compare the prediction accuracies (eg, sensitivity, specificity, and receiver operating characteristic (ROC) curve [area under the curve, AUC]) using the CNN algorithm.

In contrast to the traditionally predictive method, we use the known responses and their corresponding labels (ie, suspicious BO+ or BO−) to build a model for predicting the unknown label of the specific responses. The reason for reaching a 100% accuracy rate on the known responses and their corresponding labels in the training set is to avoid letting the CNN fail in the classification of the known responses in the future. A scheme named matching personal response scheme to adapt for the correct classification in the model (MPRSA) is designed for driving the model’s accuracy toward 100%. The way we applied the MPRSA is presented for achieving this 100% goal if the same response string is encountered in the future: the MPRSA is regarding the original responses (eg, the 20-item string coded as 9223372036854775807) that are linked to the correct label in the validation or testing set through which all cases in the training set would reach a 100% accuracy rate if the cases are present in the testing set.

#### Task 2: Validation Compared With the Training and Testing Sets

The 1002 cases were split into training and testing sets in a proportion of 70%:30%, and the former was used to predict the latter. The accuracy rates in these two sets were compared.

#### Task 3: App Detecting Burnout for a Web-Based Assessment

A 20-item self-assessment app using participant mobile phones was designed to predict nurse BO using the CNN algorithm and the model parameters [37]. The resulting classification appears on smartphones. The visual representation with binary (BO– and BO+) category probabilities is shown on a dashboard using Google Maps to display.

### Statistical Tools and Data Analysis

**Figure 2:**
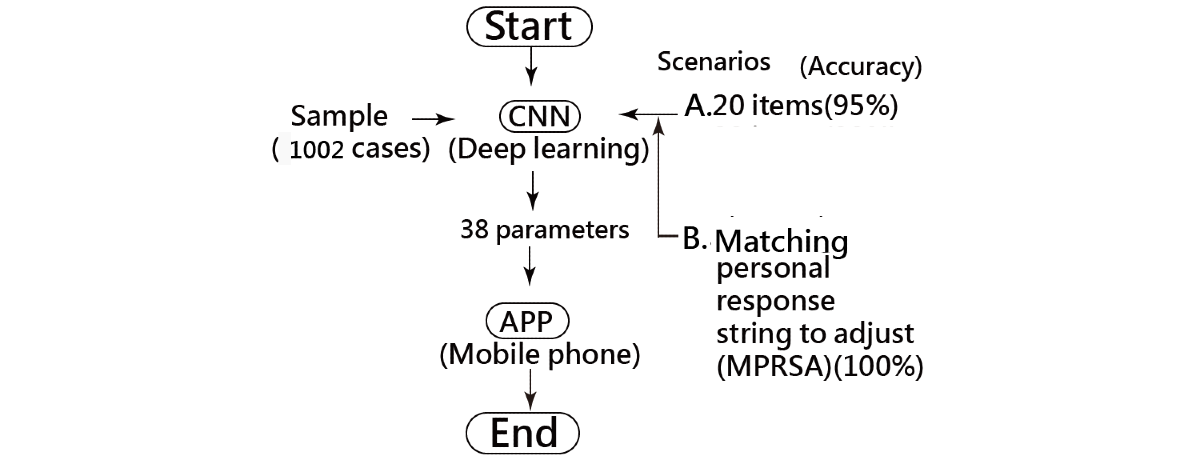
Study flowchart. CNN: convolutional neural network; MPRSA: matching personal response scheme to adapt for the correct classification in the model.

MedCalc 9.5.0.0 for Windows (MedCalc Software) was used to calculate the sensitivity, specificity, and corresponding AUC using logistic regression when the observed labels (ie, 0 for BO– and 1 for BO+) and the predicted probabilities (ie, the continuous variable in step 3 calculated by the sigmoid function in the output layer in Figure 1) were applied. A visual representation displaying the classification effect is plotted using two curves (ie, one from the left-bottom to the right-top corner denotes the success [BO+] feature and another from the left-top corner to the right-bottom side as the failure attribute). The study flowchart and the CNN modeling process are shown in Figure 2 and Multimedia Appendix 2, respectively.

## Results

### Demographic Data of Participants

The demographic data of the nurses are shown in Table 1. We can see that females accounted for 93.1% (933/1002) of the participants. Most participants had a bachelor’s (university) degree (892/1002, 89.0%). The single accounted for 59.5% (596/1002), and the married (399/1002, 39.8%). Among the nurses, 37.3% (37/1002) had work experience outside the study hospital, while 62.5% (627/1002) had none.

The highest in nurse hierarchy is N (132/1002, 13.2%), followed by N1 (134/1002, 13.4%), N2 (272/1002, 27.1%), N3 (248/1002, 24.8%), and N4 (215/1002, 21.5%). The top two job titles are nurse (797/1002, 79.5%) and leader (149/1002, 14.9%).

The average age for the sample is 32.6 (SD 7.2) years, ranging from 23 to 56. The average work experience in other hospitals reaches 15.1 (SD 28.5) months.

The workload in terms of the number of patients cared for in a week by each nurse averages 11 (SD 19.1). The mean for non-care affairs in a week reaches 4 hours (SD 5.8). The mean of nursing care is 9 (SD 2.7) hours per week. The average number of a patient cared for is 9 (SD 12.1).

**Table 1:** Demographic data of the study sample.

Variable and type		Value
**Gender, n (%)**		
	Male	69 (6.9)
	Female	933 (93.1)
**Education, n (%)**		
	Less than university	46 (4.6)
	University	892 (89.0)
	Graduate school	64 (6.4)
**Marital status, n (%)**		
	Single	596 (59.5)
	Married	399 (39.8)
	Divorced	7 (0.7)
**Work tenure, n (%)**		
	Without	627 (62.6)
	With	375 (37.4)
**Nurse hierarchy, n (%)**		
	N (<1 year experience)	133 (13.3)
	N1 (Fundamentals of Nursing)	134 (13.4)
	N2 (Critical Care in Nursing)	272 (27.1)
	N3 (Holistic Care and Teaching)	248 (24.8)
	N4 (Specialist Nursing and Research)	215 (21.5)
**Job title, n (%)**		
	Nurse	798 (79.6)
	Leader	147 (14.7)
	Assistant head nurse	30 (3.0)
	Head nurse	27 (2.7)
Age, mean (SD), range		32.6 (7.2), 23-56
Work experience outside hospital (month), mean (SD), range		15.1 (28.5), 0-180
Average hours spent in non-care affairs per week, mean (SD), range		3.9 (5.8), 0-60
Average weekly hours spent in nursing care, mean (SD), range		9.2 (2.9), 1.5-70
Average daily patient care, mean (SD), range		9.5 (12.1), 0-120

### Unsupervised Learnings Using the K-Mean Clustering

**Figure 3:**
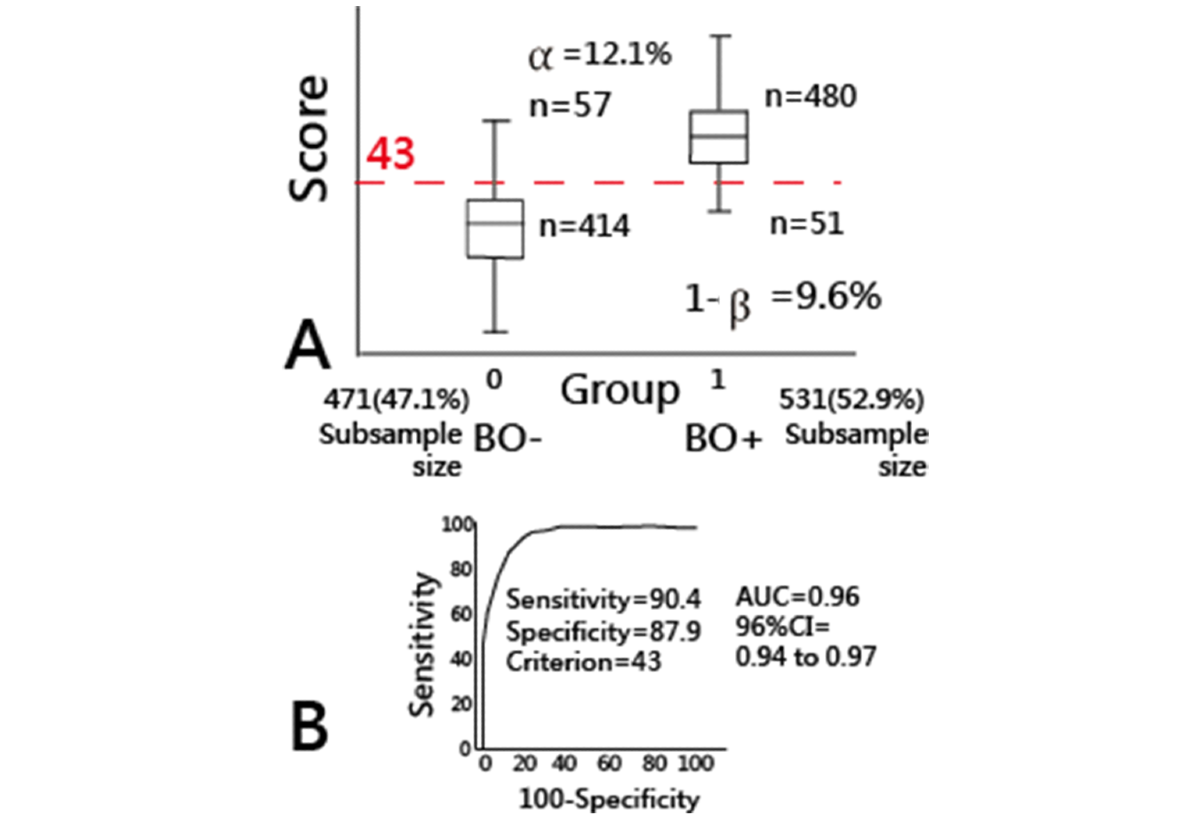
Two study groups divided by the k-mean algorithm (A) and receiver operating characteristic curve (B).

A visual representation displaying the classification effect is plotted using the box plot (Figure 3). We can see a smaller number of cases with suspicious BO– having a higher total score, and a smaller number of cases are misclassified as BO+ (12.1%) and BO− (9.6%). In contrast, the sensitivity and specificity are 90.4% and 87.9%, respectively. The cutting point is set at 43 with an AUC 0.96 (bottom, Figure 3) if the unsupervised learning approach is applied.

### Tasks to Compare the Accuracy Between Modes

#### Comparison of Prediction Accuracies in Two Modes

The 20-item model yields a higher accuracy rate (0.95) with an AUC 0.98 (95% CI 0.97-1.00) higher than that of the 20-item model with an accuracy of 0.95 and an AUC 0.97 (95% CI 0.96-0.99) based on the 1002 cases.

The MPRSA applied to the bottom pattern in Table 2 drives the model’s accuracy at 100%.

#### Validation Compared With the Training and Testing Sets

The 700-case training set with 0.96 accuracies predicts the 302-case testing set reaching an accuracy of 0.91 (Table 3).

**Table 2:** Three scenarios applied to convolutional neural network for the prediction of nurse burnout (n=1002).

Sample		True condition			
		BO+^a^	BO–^b^	BO+/row #	BO–/row #
**Scenario A (only 20 items)**					
	Positive	507	26	0.95	0.05
	Negative	24	445	0.05	0.95
**Scenario B (Scenario A and MPRSA^c^) training**					
	Positive	531	0	1.00	0
	Negative	0	471	0	1.00

^a^BO+: suspicious for burnout.

^b^BO–: not suspicious for burnout.

^c^MPRSA: matching personal response scheme to adapt for the correct classification.

**Table 3:** Training and testing effects.

Sample		True condition							
		BO+^a^		BO–^b^		BO+/row #		BO–/row #	
**Scenario A (20 items) training, n=700**									
	Positive		362		15		0.96		0.04
	Negative		10		313		0.03		0.97
**Scenario B (20 items) testing, n=302**									
	Positive		147		16		0.90		0.10
	Negative		11		128		0.08		0.92

^a^BO+: suspicious for burnout.

^b^BO–: not suspicious for burnout.

### App Detecting Burnout for a Web-Based Assessment

**Figure 4:**
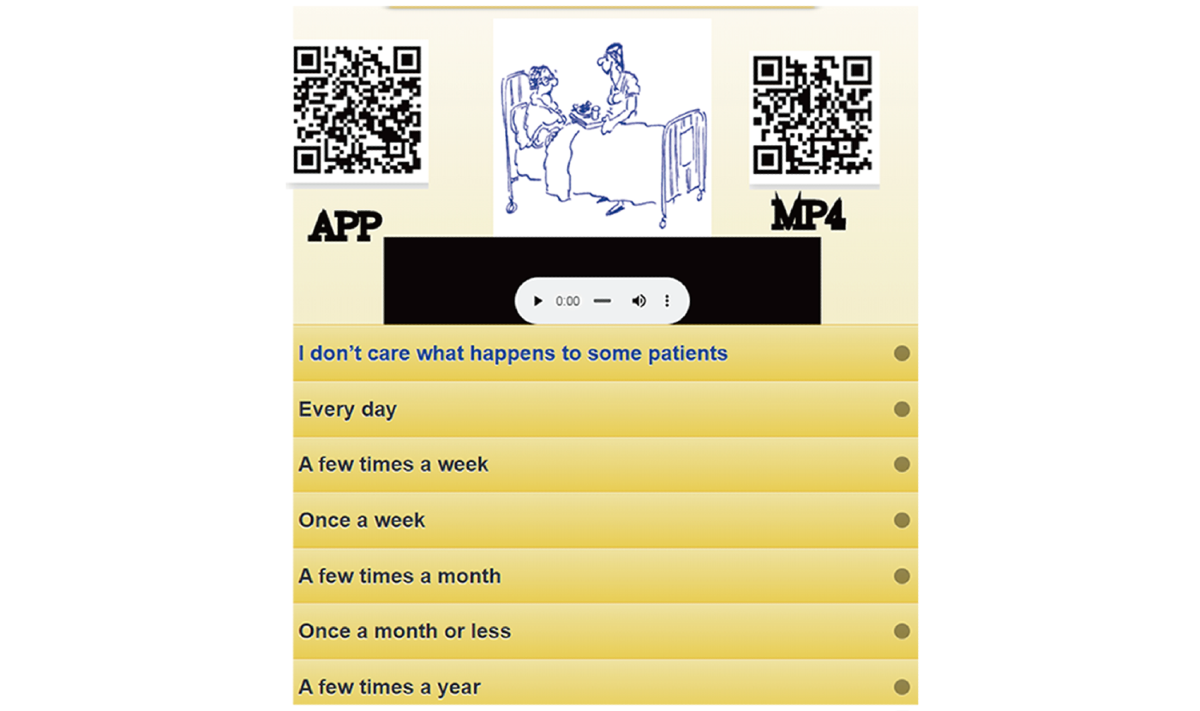
Screenshot of the mobile phone app.

An MBI-HSS app for nurses predicting BO was developed (Figure 4). Interested readers are invited to scan the QR code to practice the MBI-HSS app on their own. It is worth noting that all 38 model parameters are embedded in the 20-item CNN model for classification of either suspicious BO+ or BO− once all 20 items have been responded to.

**Figure 5:**
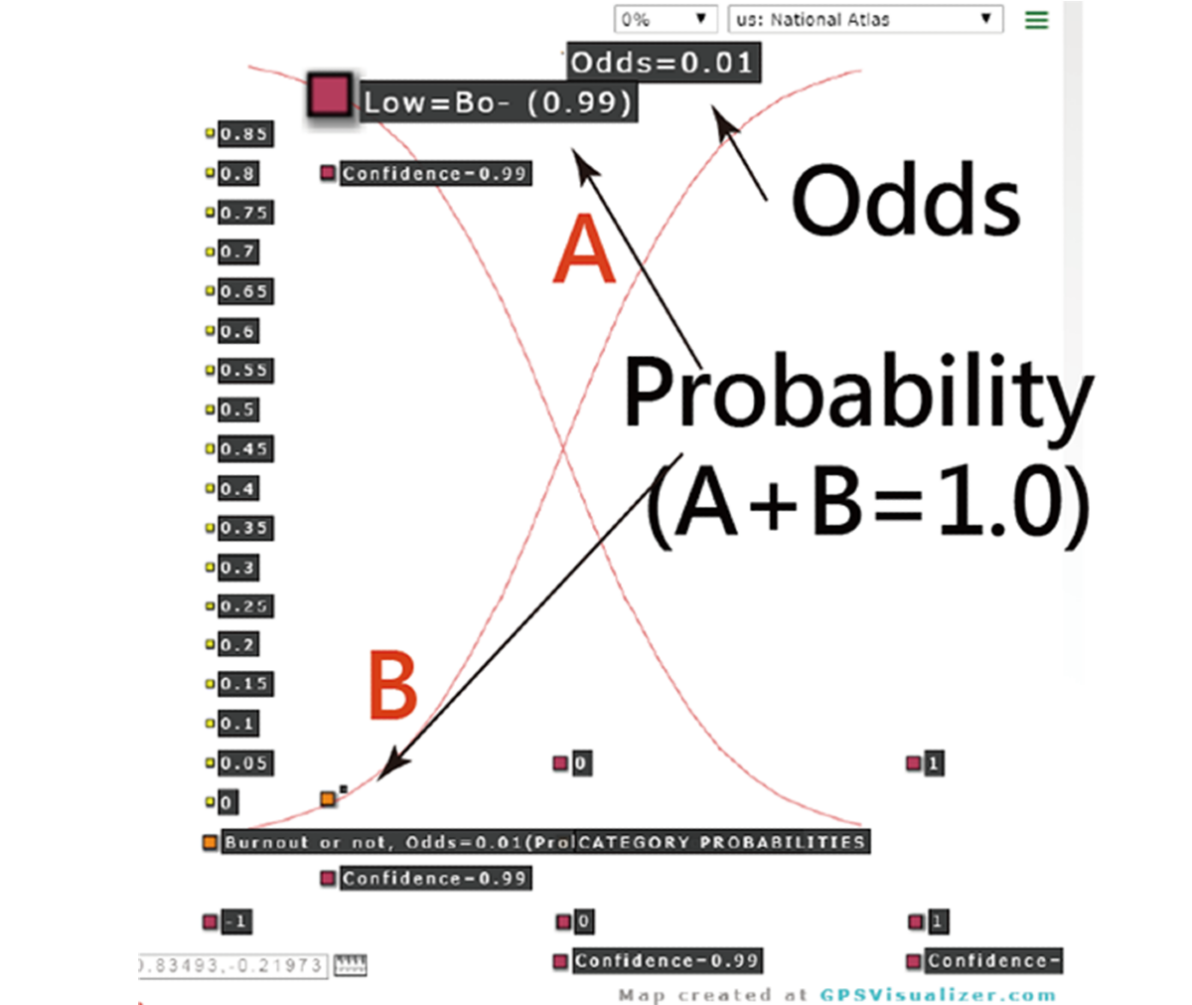
The result of assessing nurse burnout.

One resulting example is present in Figure 5, from which we can see that the BO– with a high probability (0.99) is shown on the curve of the failure from the left-top to the right-bottom corner. The sum of both probabilities (ie, BO+ and BO–) equals 1.0. The odds can be computed by the formula (p/[1–p]=0.01/0.99=0.01), indicating the nurse with an extremely low probability or tendency toward BO+.

## Discussion

### Principal Findings

We observed that (1) the 20-item model yields a higher accuracy rate (0.95; AUC 0.97, 95% CI 0.94-0.95), (2) the MPRSA drives the model’s prior accuracy at 100%, (3) the 700-case training set with 0.96 accuracy predicts the 302-case testing set reaching an accuracy of 0.91, and (4) the MBI-HSS app for nurses predicting BO has been developed and demonstrated.

The MBI-HSS is the most widely used tool for measuring BO in the world [11,14-16]. More than 1898 articles were found by searching the keywords “Maslach” and “burnout” on September 23, 2019. However, none provided an acceptable scheme to classify BO levels (ie, BO+ and BO− or low, moderate, and high).

Maslach and Jackson [13] provided a cutting point scheme (ie, 54 for emotional exhaustion, 48 for personal accomplishment, and 30 for depersonalization; around 40=132/3*20/22) approximately equal to 43 (based on 20 items using subscale total scores; see Figure 3) in this study. Although Schaufeli and Van Dierendonck [23] doubted that the cutting points proposed by Maslach and Jackson [13] were arbitrary only on the assumption of an equal sample size across the levels (ie, high, moderate, and low), our cutting point at 43 is derived through the k-mean clustering.

However, no matter which cutting point scheme is applied, that of Maslach and Jackson [13] or this study (eg, in Figure 3), misclassifications must exist due to their type I (α) and II (1–β) errors. In contrast, the CNN predictive model combined with the MPRSA mentioned in Methods regarding task 1 (100% accuracy rate is required) can minimize the type I and II errors toward zero (eg, Table 2), which is one of the features of this study.

### Implications and Future Work

CNN can improve prediction accuracy (up to 7.14%) [28]. In this study, sensitivity and specificity have been improved. So far, we have not seen anyone using the CNN approach to predict nurse BO in the literature, which is a breakthrough, and the first feature of this study.

Over 708 articles have been found using the keyword “convolutional neural network” (Title) searched in PubMed Central on September 23, 2019. None used Microsoft Excel to perform the CNN. The interpretations for the CNN concept and process or the parameter estimations are shown in Figure 1, Multimedia Appendix 2 and 3, and in the app [38], which is the second feature of this study.

Using Microsoft Excel to perform CNN is the third feature of this study (Multimedia Appendix 1), which was rarely seen applicable in the literature.

Because the principle for concerning more with the vital few and less with the trivial numerous is emphasized in the quality control process, we propose the MPRSA as the fourth feature. We incorporated the original responses into the model to let the label be correctly classified by the CNN, through which all cases with a false prediction in the training set would be adjusted as a true prediction, reaching a 100% accuracy rate if the cases reoccur in the testing set.

Furthermore, the curves of category probabilities based on the Rasch rating scale model [39] are shown in Figure 4. The binary categories (eg, success and failure on an assessment in the psychometric field) have been applied in health-related outcomes [40-44]. However, none provided the animation-type dashboard showing on Google Maps, as we did in Figure 4.

### Strengths

It is easy to set up the nurse BO online assessment if the designer uploads relevant and appropriate audio and visual files to the corresponding questions of the database. We applied the CNN algorithm along with the model’s parameters to design the routine on an app that is used to detect BO risk for nurses in hospitals (Figure 4), which has never been seen before for the MBI-HSS [13] implemented on mobile phones.

As with all forms of web-based technology, advances in health communication technology are rapidly emerging [29]. Mobile online BO assessment is promising and worth considering in many fields of health assessment. An online BO assessment (Figure 4) can be applied to inform examinees quickly about when and whether they should take actions or follow up to see a psychiatrist and how to improve their behaviors and attitudes given that their lifestyle is not changed [4]. The online BO assessment is promising, and it is worth using for promoting nurses’ health literacy by using the animation-type assessment on smartphones. Interested readers are recommended to scan the QR codes on Figure 4, one for the app and another for the MP4, and see the details about responding to questions and the real experience on answering the 20-item MBI-HSS for a website assessment.

The CNN module on Microsoft Excel is unique and innovative (Multimedia Appendix 1). Users who are not familiar with the CNN software (eg, Python) can apply our Excel Visual Basic for Applications module to conduct CNN-related research in the future. The module is not limited to the binary classification. The multiclassification module can be done by adding the layers on CNN. That is, two categories require two input layers and two pooling layers. Similarly, three categories need three input layers and three pooling layers (Figure 1 and Multimedia Appendix 1 and 2). Any other types of self-assessment, such as work bullying, depression, and dengue fever, can apply the CNN model to predict and classify the levels of harmfulness and disease in the future.

### Limitations and Suggestions

Our study has some limitations. First, although the psychometric properties of the 20-item MBI-HSS have been validated for measuring nurse BO in Taiwan [3] after removing item 14 (I feel I am working too hard on my job) and item 22 (I feel patients blame me for some of their problems), there is no evidence that supports that the 20-item MBI-HSS is suitable for nurses in other regions. We recommend additional studies using their own k-mean algorithm and the CNN model to estimate the parameters and see the difference (eg, the cutting point at 43 in Figure 3).

Second, we have not discussed any improvement in predictive accuracy. For instance, whether other featured variables (eg, the mean, SD, and Lz index [44,45]) applied to the CNN model can increase the accurate rate is worthy of further study. Future studies are needed to look for other variables that can improve the power of the model prediction.

Third, the study was based on previously published [3] research using the 20-item MBI-HSS. All of the data were sampled from similar health care settings. If any environment or condition is changed (eg, other professionals or workplaces), the result (eg, the model’s parameters) must be different from this study.

Fourth, the MBI-HSS is a three-dimensional construct. Usually, the item difficulties should be first calibrated by using the Rasch ConQuest software [46]. The CNN model [47] can ignore the issue of dimensionality and gain a favorable prediction effect that should be verified and ensured in the future.

Finally, the study sample was taken from Taiwanese data in a nurse survey. The model parameters estimated for the MBI-HSS Chinese version are only suitable for the Chinese (particularly for Taiwanese) society in health care settings. Generalizing these BO assessment findings (eg, the cutting point at around 43; see Figure 3) might be somewhat limited and constrained because the sample merely consisted of nurses working for inpatients. Additional studies are needed to reexamine whether the psychometric properties of the BO assessment are similar to that of other worksites in (or out of) a hospital.

### Conclusion

We illustrate features and contributions in this study: (1) CNN performed in Microsoft Excel, (2) MPRSA applied to increase the model’s prior prediction accuracy, (3) an online app demonstrated to display results using a visual dashboard on Google Maps, and (4) the category probability curves based on Rasch rating scale model first observed in the CNN prediction model. The novelty of the app with the CNN algorithm improves the predictive accuracy of nurse BO. It is expected to help nurses self-assess job BO at an early stage in the future.

## Appendix

Multimedia Appendix 1 Study dataset.

Multimedia Appendix 2 Convolutional neural network to interpret Figure 1.

Multimedia Appendix 3 Mp4 for convolutional neural network performed in Excel.

## Abbreviations

AUC: area under the curve
BO: burnout
CNN: convolutional neural network
MBI-HSS: Maslach Burnout Inventory–Human Services Survey
MPRSA: matching personal response scheme to adapt for the correct classification
PA: personal accomplishment
ROC: receiver operation characteristic
