# Supplementary material for: An App Developed for Detecting Nurse Burnouts Using the Convolutional Neural Networks in Microsoft Excel: Population-Based Questionnaire Study
Source: JMIR Med Inform. 2020 May 7;8(5):e16528. doi: 10.2196/16528 (PMC7243132; doi:10.2196/16528)
Supplement: Multimedia Appendix 2 [file medinform_v8i5e16528_app2.docx]

1. **Preparing the case responses**:

One case (BO+ or BO-) is defined as {0,1} or{1,0}. His/her responses(e.g., {0,1,1,….., 01}) are sequentially assigned into the 6$\times6 metric$ in the input layer, see panel B in Figure 1.


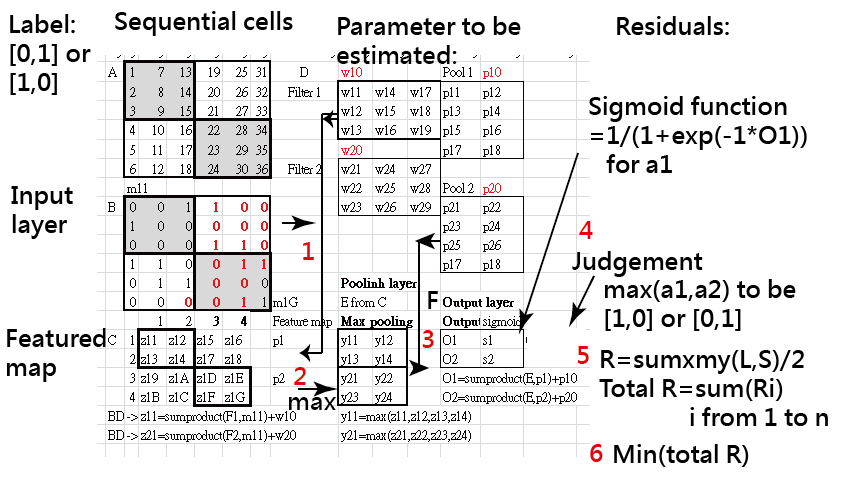


Figure 1 Interpretation for CNN algorithm in Micro-soft Excel

1. **Building the model**:

Two filters are assigned in this burnout example due to two categories (BO+ or BO-) labeled here. A total of 38 parameters are required to estimate by the CNN model, including two filters(i.e., each with 9 parameters and1 biases denoted by w10 and w20) and two pools(i.e., each with 8 parameters and 1 biases denoted by p10 and p20), see panel D in Figure 1.

1. **Step 1 on the convolution layer**:

Panel B in Figure 1 resembles the 6x6 input image. Two featured maps(or say convoluted map) in the Convolutional Layer are obtained at the panel C in Figure 1 by performing a matrix multiplication operation on its filter and the portion m(e.g., m11 or m1G) of the image over which the filter is hovering.

We have selected Filter(or say Kernel in CNN term) as a 3x3 matrix, see the panel D. The Filter shifts 16 times because of Stride Length = 1 (i.e., move one step for each hovering). Sixteen stride values for one specific filter are generated on the corresponding featured map, see the panel C in Figure 1. Each element is valued by the equation[=sumproduct(F, m)+bias, where F denotes the filter, m represents the portion of the image over which the filter is hovering, and bias stands for the corresponding filter as w10 or 20 in panel D, see Figure 1].

1. **Step 2 on the pooling layer**:

The upper four elements(from y11 to y14 corresponding to the Filter 1) are obtained by selecting the maximum of the four elements in each 2$\times$2 matric in panel E. Similarly, the lower four elements(from y21 to y24) are yielded by taking out the maximum of the four elements in each 2$\times$2 matric from another featured map corresponding to the Filter 2.

**Step 3 on the pooling layer**:

The 4$\times$2 matric in the pooling layer is cooperated with the corresponding polling parameters in panel D for generating the output values(i.e., O1 and O2 in panel F) through the equation(=sumproduct(the pooling layer, the pooling combo 1 or 2)+bias(i.e., p10 or p20 in panel D). Usually, we apply a sigmoid function(=1/(1+exp(-1*output value))) to let the output values ranged from 0 to 1.

**Step 4 for classifying the label**:

The maximum of the output values (i.e., a1, a2 in panel F) is redirected to the predicted label(i.e., BO+ as [0,1] or BO- as [1,0]).

**Step 5 for computing the case residual**:

The model residual is calculated by the least squared equation(=$\sum_{i-1}^{2} {(O_{i}-E_{i})}^{2},$ where Oi is the observed label and Ei denotes the predicted label). In MS Excel, the function(=sumxmy(m(observed label), m(predicted label))/2, m() represents the matrix of observed or predicted labels) is applied to compute the case residual.

**Step 6 for computing the total model residual**:

The objective function for optimizing the model is to minimize the model residual(=$\sum_{i=1}^{n} R_{i},$ where R denotes the case residual).

**(C)Optimizing the model**:

When all the training cases are put into the model using the CNN algorithm in MS Excel, the Solver Add-in is applied to estimate those 38 parameters when all initial parameters were assigned by standardized normal random numbers(i.e., mean=0 and standard deviation =1) in panel D of Figure 1, see Multimedia Appendix 1 and 2.
